# Essays and Orations

**Published:** 1831

**Authors:** 


### ESSAYS AND ORATIONS. By Sir *Henry Halford*.

### I. ON TIC DOULOUREUX.

AMONG the Essays and Orations lately published by the distinguished Baronet, the first (not previously printed) is on Tic Douloureux, in which some peculiar doctrines are broached respecting the pathology, or rather the etiology of that terrible disease. The principal symptoms are enumerated in Sir Henry's usual elegant language, but these we may pass over. The experienced author believes that the milder forms of TIC, usually denominated neuralgiæ, and seated in various nerves of the body besides those of the fifth pair, generally depend on some derangement of the digestive organs, and usually give way to treatment directed to those sources; but the severe TIC of the trigemini does not yield to any particular treatment with which we are yet acquainted, though it may be mitigated by attention to the general health. That the seat of pain is not the seat of disease, is proved by the failure of attempts to cut off the communication of the suffering nerves with the brain.

"May I venture (says Sir H.) to throw out an opinion, founded on the observations with which my experience has furnished me, that the disease is connected with some preternatural growth of bone, or a deposition of bone in a part of the animal economy where it is not usually found, in a sound and healthy condition of it, or with a diseased bone?

The following cases have occurred to me, and seem to give a degree of probability to this surmise; and I throw it out for the consideration of the profession, in order that a number of facts may be collected from which a safe inference at length can be drawn.

I. A lady, forty years of age, suffered under the violent form of tic douloureux, at Brighton, notwithstanding the careful attention and skill of a very judicious physician there. On returning to town it was observed that the rending spasms, by which the disease is marked, were frequently preceded by an uneasiness in one particular tooth, which exhibited, however, no signs of unsoundness; but the constancy of this symptom was enough to justify the extraction of the tooth in this instance (though the failure of this expedient to afford relief in general does not encourage recourse to the operation), and, on its being drawn, a large exostosis was observed at the root of the tooth; and the lady never suffered more than very slight attacks, and those very seldom afterwards.

II. The Duke of G. was attended by Dr. Baillie and myself for six weeks, under this disease, in its most marked and painful form, without deriving benefit from our prescriptions. At length we thought it best to advise him to repair to the sea-coast, in hopes of renovating his shattered system by taking bark there. After he had sojourned a month by the sea-side, a portion of bone exfoliated from the antrum Highmorianum, and the Duke recovered immediately, and has never suffered the disease since. The bone had been hurt probably by a fall from his horse which the Duke had met with some months before.

III. The late Earl of C. underwent martyrdom by this disease, and excited the warmest sympathy of his friends by the agonies he sustained for many years. He submitted to the operation for the division of several branches of the fifth pair of nerves repeatedly, by Sir Edward Home and by Mr. Charles Bell, without obtaining more than mere temporary relief. At length he was seized by apoplexy, and lay insensible for some days, and in great peril from the attack, but finally recovered. After the apoplexy, the paroxysms of the tic douloureux became less frequent and less severe, and were administered to satisfactorily by an ingenious physician, who wrote his inaugural exercise on the disease. For the last year or two of his life his lordship had ceased to suffer from the tic, and died at an advanced age without any marked malady. His head was not examined after death, and therefore we are left to conjecture only what might have been the immediate cause of his former sufferings. Whilst I attended him he underwent repeated exfoliations of the alveolar processes of the teeth, which I thought occasioned his torment; and to account for the cessation of the complaint, I supposed that these efforts to throw off diseased portions of bone might have ceased, or that the apoplexy had disqualified the nerves for suffering so exquisitely; but there might have been besides, as some later instances have made probable, disease in the bones of the head.

IV. The late Dr. P. fell a sacrifice to this dreadful disease, after sustaining its tortures, for some years, with a constancy which attracted all our pity and esteem, and died at last under apoplexy.

No assistance which the experience of any of us could afford him, gave him relief or controlled the violence of the attacks. On examining his head after death, there was found an unusual thickness of the os frontis, where it had been sawn through above the frontal sinuses, and at its juncture with the parietal bones. There was discovered also in the falciform process of the dura mater, at a little distance from the crista galli, a small osseous substance, about three-eighths of an inch in length, rather less in breadth, and about a line in thickness. The vessels of the pia mater were turgid with blood, and about an ounce of fluid occupied the ventricles. I lamented that the frontal sinuses had not been examined, for I remember he replied to a question which I once put to him, as to his ever having experienced any suppuration within any bony cavity, that he had twice suffered suppuration in the frontal sinuses.

Dr. P. had submitted with great patience to a division of several branches of the fifth pair of nerves, under the judicious operation of Sir Astley Cooper, who, on my mentioning to him the notion I entertained of the cause of tic douloureux, was so obliging as to show me the skull of a person who had died of this disease in the country. The internal surface of the frontal bone is a perfect rock-work." 44.

The foregoing cases fell under the immediate observation of the author. The following was communicated to him by a physician of eminence in the country. The patient was a lady advanced in life. At the age of 65 she was attacked with exquisite pain in the branches of the fifth pair of nerves, on the right cheek, nose, and temple, the tortures of which were unbearable. For nearly ten years the paroxysms continued to recur with more or less of intermission; and the operation of dividing the supra-orbital branch of the nerve was succeeded by an alleviation of pain during five months. They then returned. Various plans of treatment were employed; but with little success. Carbonate of iron and valerian gave most relief. Of the former she took *twenty-seven pounds*—a quantity that might have satisfied even our good friend Dr. Elliotson. Her intellect was unimpaired, and her general health did not appear affected. She was free from pain during the last six months of her life, which terminated in apoplexy.

"The head was opened after death, and an enormous thickening was observed of the frontal, ethmoidal, and sphenoidal bones, in one part to the extent of half an inch; and the anterior lobes of the brain were curiously moulded and indented by the thickened bone. There was thickening also of the whole of the cranium, but not to so great a degree any where as in the parts which have just been named." 46.

The paper terminates with some observations on sympathy which we need not analyze. Sir Henry's observations offer a strong probability that ONE cause of severe neuralgic affections may be attributed to bony excrescences or osseous disease. But when we reflect on the immense mass of similar diseases of bone, where no TIC DOULOUREUX is the result, we must still deplore the darkness in which we are placed respecting this terrible disease!

### II. Popular and Classical Illustrations of Insanity.

This paper, which was also read at the College of Physicians, is prefaced by Shakespeare's test of madness.

> "——— Ecstacy !
> My pulse as yours doth temperately keep time,
> And makes as healthful music. It is not madness
> That I have utter'd: bring me to the test,
> And I *the matter will re-word, which madness*
> *Would gambol from.*"
>
> Hamlet, Act. iii., Scene 4.

To prove the correctness of the poet's test, Sir Henry adduces a case which occurred in his own practice, in the month of January, 1829.

"A gentleman of considerable fortune in Oxfordshire, about thirty-five years of age, sent for his solicitor to make his will. He was in habits of strict friendship with him, and stated that he wished to add five hundred pounds a year to his mother's jointure, if she got well, she being then (to the knowledge of the solicitor and himself only) confined as a lunatic; to make a provision for two natural children; to leave a few trifling legacies; and then, if he died childless, *to make him, the solicitor, his heir.* His friend expressed his gratitude, but added that he could not accept such a mark of his good opinion, until he was convinced that it was his deliberate judgment so to dispose of his property, and that decision communicated to him six months afterwards.

In about six weeks time the gentleman became deranged, and continued in such a state of excitement for a whole month, (during which he was visited constantly by Sir George Tuthill and myself,) as to require coercion every day. At the expiration of that time he was composed and comfortable. But his languor and weakness bore a proportion to his late excitement, and it was very doubtful whether he would live. On entering his room one day, to my question how he found himself, he answered,—'Very ill, Sir; about to die; and only anxious to make my will first.' This could hardly be listened to under his circumstances, and he was persuaded to forego that wish for the present. The next day he made the same answer to the same question, but in such a tone and manner, as to extort from common humanity, even at the probable expense of future litigation, an acquiescence in his wish to disburthen his mind. The solicitor was sent for, and, having been with him the preceding evening, met us, at our consultation in the morning, with a will prepared according to the instructions he had received *before the attack of disease, as well as to those given the last night.* He proposed to read this to the gentleman in our presence, and that we should witness the signature of it, if we were satisfied that it expressed clearly his intentions. It was read, and he answered, 'yes,'—'yes,'—'yes,' distinctly to every item, as it was deliberately proposed to him. On going down stairs with Sir George Tuthill and the solicitor, to consider what was to be done, I expressed some regret that we, the physicians, had been involved in an affair which could hardly be expected to terminate without an inquiry in a court of law, in which we must necessarily be called upon to justify ourselves for permitting this good gentleman, under such questionable circumstances, to make a will. It occurred to me then, to propose to my colleague to go up into the sick room, to see whether our patient could *re-word* the matter, as a test, on Shakspeare's authority, of his soundness of mind. He repeated the clauses which contained the addition to his mother's jointure, and which made provision for the natural children, with sufficient correctness; but he stated that he had left a namesake, though not a relation, ten thousand pounds, whereas he had left him five thousand pounds only; and there he paused. After which I thought it proper to ask him, to whom he had left his real property, when these legacies should have been discharged,—in whom did he intend that his estate should be vested after his death, if he died without children? 'In the heir at law, to be sure,' was the reply. Who is your heir at law? 'I do not know.'

Thus he 'gambolled' from the matter, and laboured, according to this test, under his madness still." 59.

This gentleman died intestate, of course, four days afterwards. The "gambolling" above-mentioned, was no doubt a sufficient test of some mental aberration at the time; and we think there can be as little doubt that the gentleman, on close examination, would have been found incoherent on many other subjects besides that of the WILL.

After a well-merited eulogy on Shakespeare, as the poet that holds up to his readers a faithful mirror of manners and of life, Sir Henry informs us that it has twice occurred to him to find the portraits of madness, as drawn by Horace, exemplified to the life.

" One case, that of the gentleman of Argos, whose delusion led him to suppose that he was attending the representation of a play, as he sat in his bedchamber, is so exact, that I saw a person of exalted rank under those very circumstances of delusion, and heard him call upon Mr. Garrick to exert himself in the performance of Hamlet. The passage of Horace to which I allude is in the second epistle of the second book, and is the more curious as it specifies distinctly that it was upon this one point only that the gentleman was mad. I will give you the passage:

———————— Fuit haud ignoblis Argis,
Qui se credebat miros audire tragœdos,
In vacuo lætus sessor plausorque theatro;
Cætera qui vitæ servaret munia recto
More; bonus sane vicinus, amabilis hospes,
Comis in uxorem, posset qui ignoscere servis,
Et signo læso non insanire lagenæ:
Posset qui rupem et puteum vitare patentum.
&c. &c. *Epist.* lib. ii. 2. 128.

In another well known case, which justified the Lord Chancellor's issuing a writ *de lunatico inquirendo*, the insanity of the gentleman manifested itself in his appropriating every thing to himself, and parting with nothing. When strongly urged to put on a clean shirt, he would do it, but it must be over the dirty one; nor would he put off his shoes when he went to bed. He would agree to purchase any thing that was to be sold, but he would not pay for it. He was, in fact, brought up from the King's Bench prison, where he had been committed for not paying for a picture valued at fifteen hundred pounds, which he had agreed to buy; and in giving my opinion to the jury, I recommended it to them to go over to his house, in Portland-place, where they would find fifty thousand pounds worth of property of every description; this picture, musical instruments, clocks, baby-houses, and baubles, all huddled in confusion together, on the floor of his dining room. To such a case what could apply more closely than the passage—

> Si quis emat citharas, emptas comportet in unum,
> Nec studio citharæ, nec Musæ deditus ulli;
> Si scalpra et formas, non sutor; nautica vela,
> Aversus mercaturis: delirus et amens
> Undique dicatur merito. Hor. Sat. lib. ii. 3. 104.

I need not add that the jury found the gentleman insane." 63.

Human nature, in fact, has been always the same; and the descriptions of it, as drawn by the ancient poets are, at this day, as correct as when they were originally drawn. Sir Henry is of opinion, that if the physician were to collect and apply the brief notices of various disorders, which have been thrown out by the great poets of antiquity, "he might not only illustrate the truth of the descriptions drawn by those accurate observers of Nature, *but derive from them some useful hints to assist him in his own observation of disease.* We much doubt whether the time consumed in searching out these faithful descriptions of diseases among ancient poets, would be so well disposed as in observing the originals themselves. The physician has the same senses and means of recognizing diseases as the poets had—why, then, have recourse to the copy, when the original portrait is before him? We are ready to grant, however, that these illustrations and researches are elegant and dignified pursuits for a leisure hour, and especially where the mind is strongly imbued (as is Sir Henry Halford's) with classical literature and modern knowledge.

